# Editorial: Advances in AI methods for computational pathology

**DOI:** 10.3389/fmed.2022.974857

**Published:** 2022-09-21

**Authors:** Xin Qi, Jun Kong, Fuyong Xing, Guotai Wang, Zhuotun Zhu

**Affiliations:** ^1^Eisai, Nutley, NJ, United States; ^2^Department of Mathematics and Statistics, Georgia State University, Atlanta, GA, United States; ^3^Department of Biostatistics and Informatics, University of Colorado Denver, Aurora, CO, United States; ^4^School of Mechanical and Electrical Engineering, University of Electronic Science and Technology of China, Chengdu, China; ^5^Department of Radiology and Radiological Science, Johns Hopkins University, Baltimore, MD, United States

**Keywords:** deep learning, computational pathology, multi-modality, convolutional neural networks, whole-slide image analysis, artificial intelligence

This special edition has received five abstracts and four manuscripts. Four research manuscripts have been accepted for publication in Frontiers in Medicine—Pathology.

Since the emergence of deep learning technology, the way and the extent of extracting signature histology features from histology whole-slide images (WSI) for clinical decision support have been significantly changed. The manuscript of “*Deep Learning-Based Pathology Image Analysis Enhances Magee Feature Correlation with Oncotype DX Breast Recurrence Score*” is a good example where deep learning models are trained to extract such histology features as tumor cell number, tumor-infiltrating lymphocytes (TIL) number variance, and nuclear grades from WSIs. Due to the powerful computation ability, deep learning can extract these histology features from the entire tissue regions in WSIs, preserving the information intactness and avoiding sampling bias. The resulting WSI-derived features, when combined with the state-of-the-art Magee features, are shown to be beneficial to oncotype DX recurrence score prediction for patients with estrogen receptor-positive breast cancer, enabling an avenue to predict chemotherapy benefits in such patient populations. This study suggests there is a wealth of clinically relevant information embedded in WSIs that require better computational tools for analyses. Deep learning can be a promising tool in enabling such information extraction for digital pathology. Additionally, the notion from this paper of combining WSI-derived histology features with other clinical and genomic information for better clinical support is valuable, as the same concept can be applied to other diseases.

Not only tissue and cellular level features, but also molecular signature can be quantified from WSIs. The manuscript of “*Study on Molecular Information Intelligent Diagnosis and Treatment of Bladder Cancer on Pathological Tissue Image*,” proposes to analyze the molecular subtypes, p. 53, and PD-L1 status of bladder cancer from pathological images. Three convolutional neural networks (CNNs) are independently trained and evaluated on 119 cases of MIBC WSIs with segmented tumors. The analysis results on three tasks are promising and the system can potentially reduce the clinical workload for doctors. The clinical significance and impact of this work can be further enhanced with external validations using multiple institutional datasets in the future.

One obstacle in deep learning for computational histopathology is the high computational cost, in large part due to the large histopathological image resolution and number of parameters in CNNs. To partially address this issue, the manuscript of “*Building Efficient CNN Architectures for Histopathology Images Analysis: A Case-Study in Tumor-Infiltrating Lymphocytes Classification*” demonstrates new techniques to reduce the network size for a lower computational cost during inference. The challenge for network simplification is to reduce the computational cost while maintaining its performance. Despite recent investigations on leveraging some existing techniques such as network pruning, sparsification, and quantization, studying extremely efficient networks remains an important research direction in deep learning for future research.

With the development of deep learning in computational pathology, the state-of-the-art CNNs with transfer learning can be used to solve universal expert-level pathological image classification tasks. This has been demonstrated in the manuscript of “*Deep Learning-Based Universal Expert-Level Recognizing Pathological Images of Hepatocellular Carcinoma and Beyond*,” where a deep learning-based model is developed for histological image classification. Specifically, a pre-trained CNN, i.e., ResNet-34, is fine-tuned by training only the last few layers of the network with image data from one institution. The trained CNN model is tested with datasets from another imaging center. Developers apply the model to classify hepatocellular carcinomas (HCCs) from non-HCCs, and compare the performance of model to human experts. It suggests that the CNN model can produce a comparable classification performance to that of experienced human experts. The deep learning model is also evaluated on two other histological image datasets, i.e., colorectal cancer and breast invasive ductal carcinoma, with good classification performance. This work suggests that deep learning applications to diagnoses and treatment recommendations are a promising area for future investigations.

## Author contributions

XQ, JK, FX, GW, and ZZ: reviewing and editing. All authors contributed to the article and approved the submitted version.

## Conflict of interest

Author XQ was employed by Eisai. The remaining authors declares that the research was conducted in the absence of any commercial or financial relationships that could be construed as a potential conflict of interest.

## Publisher's note

All claims expressed in this article are solely those of the authors and do not necessarily represent those of their affiliated organizations, or those of the publisher, the editors and the reviewers. Any product that may be evaluated in this article, or claim that may be made by its manufacturer, is not guaranteed or endorsed by the publisher.

